# Supplementary material for: The lipoprotein-associated phospholipase A2 inhibitor Darapladib sensitises cancer cells to ferroptosis by remodelling lipid metabolism
Source: Nat Commun. 2023 Sep 15;14:5728. doi: 10.1038/s41467-023-41462-9 (PMC10504358; doi:10.1038/s41467-023-41462-9)

Supplementary Information

Darapladib, a lipoprotein-associated phospholipase A2 inhibitor, sensitises cancer cells to ferroptosis by remodelling lipid metabolism

Mihee Oh, Seo Young Jang, Ji-Yoon Lee, Jong Woo Kim, Youngae Jung, Jiwoo Kim, Jinho Seo, Tae-Su Han, Eunji Jang, Hye Young Son, Dain Kim, Min Wook Kim, Jin-Sung Park, Kwon-Ho Song, Kyoung-Jin Oh, Won Kon Kim, Kwang-Hee Bae, Yong-Min Huh, Soon Ha Kim, Doyoun Kim, Baek-Soo Han, Sang Chul Lee, Geum-Sook Hwang, Eun-Woo Lee

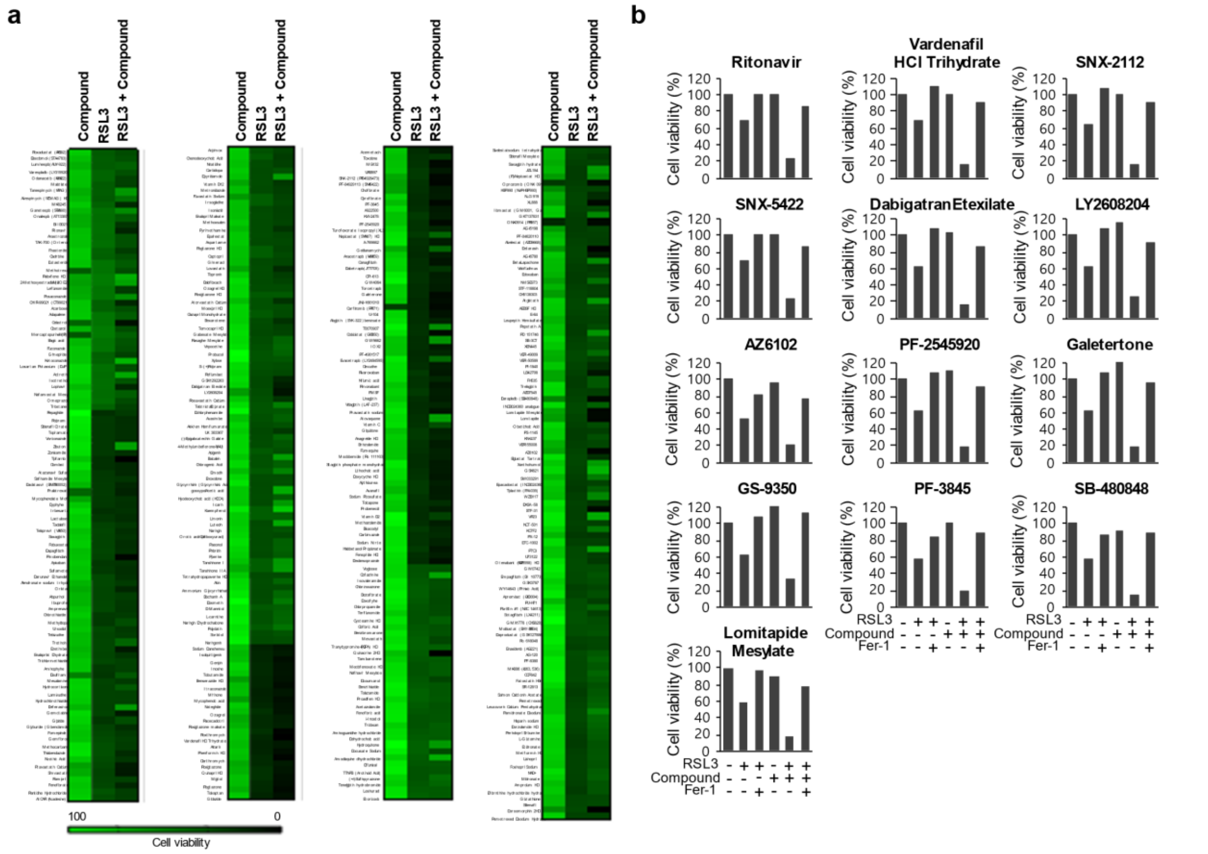

Supplementary Fig. 1. Identification of ferroptosis-modulating compounds by metabolic library screening. (a) Heatmap data indicating the viability of cells treated with the compounds at 10 µM alone or in combination with 0.5 µM RSL3. (b) Representative compounds that promote RSL3-induced ferroptosis in Hs746T cells.

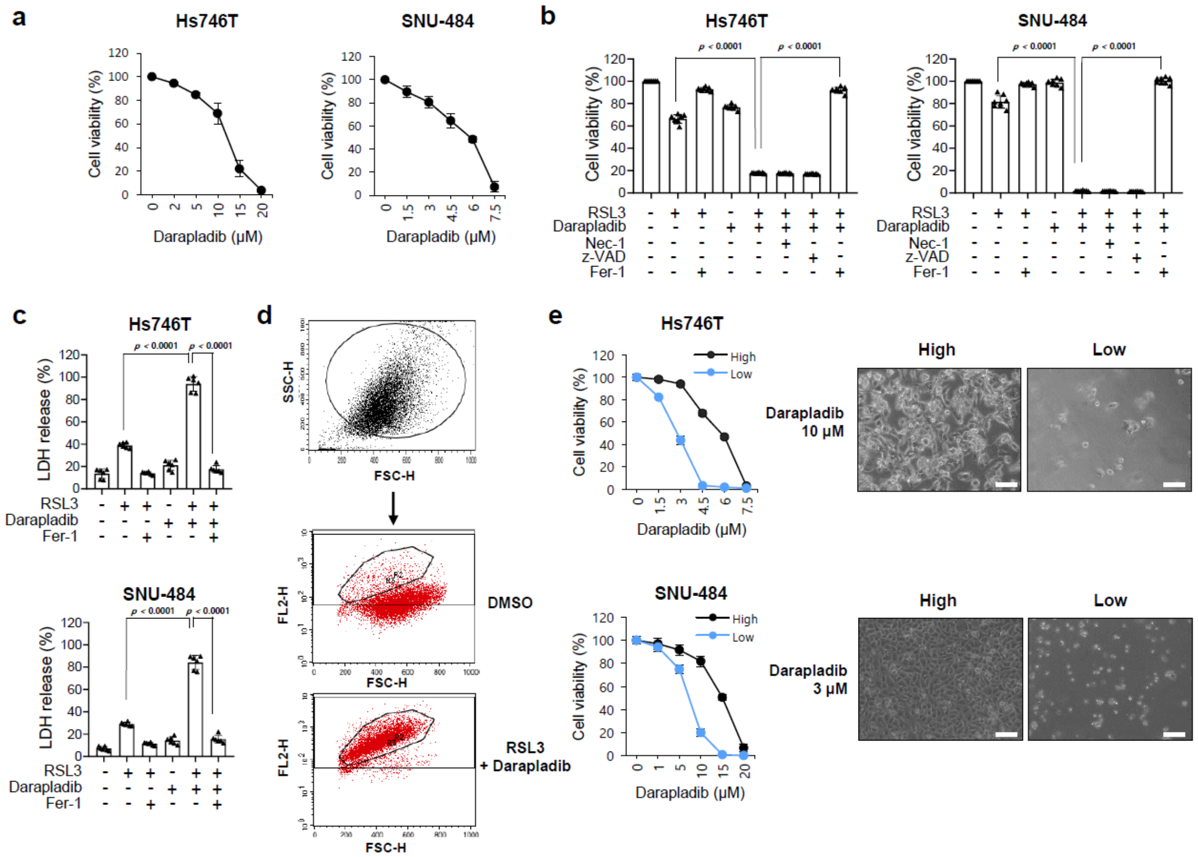

Supplementary Fig. 2. Darapladib promotes RSL3-induced ferroptosis.

(a) Relative viability of Hs746T and SNU-484 cells treated with various concentrations of darapladib for 20 h. The data are presented as the means ± SDs (n = 3 independent experiments). (b) Relative viability of Hs746T and SNU-484 cells treated with 0.2 µM RSL3 and/or 2 µM darapladib in the presence or absence of 1 µM Fer-1, zVAD-fmk (z-VAD, 20 µM) or necrostatin-1 (Nec-1, 30 µM). The data are presented as the means ± SDs (n = 8 independent experiments, the significance of the results was assessed using a two-tailed Student' *t* test). (c) LDH levels of Hs746T and SNU-484 cells treated with 0.2 µM RSL3 and/or 2 µM darapladib in the presence or absence of 1 µM Fer-1 for 20 h. The data are presented as the means ± SDs (n = 6 independent experiments, the significance of the results was assessed using a two-tailed Student' *t* test). (d) Example gating strategy for flow cytometry analysis of PI uptake in cells. (e) Relative viability of Hs746T and SNU-484 cells plated at high and low densities and treated with various concentrations of darapladib. Images of cells treated with the indicated concentration of darapladib are shown. The data are presented as the means ± SDs (n = 6 independent experiments). Scale bar, 200 um. Exact p values provided as source data. Source data are provided as a source data file.

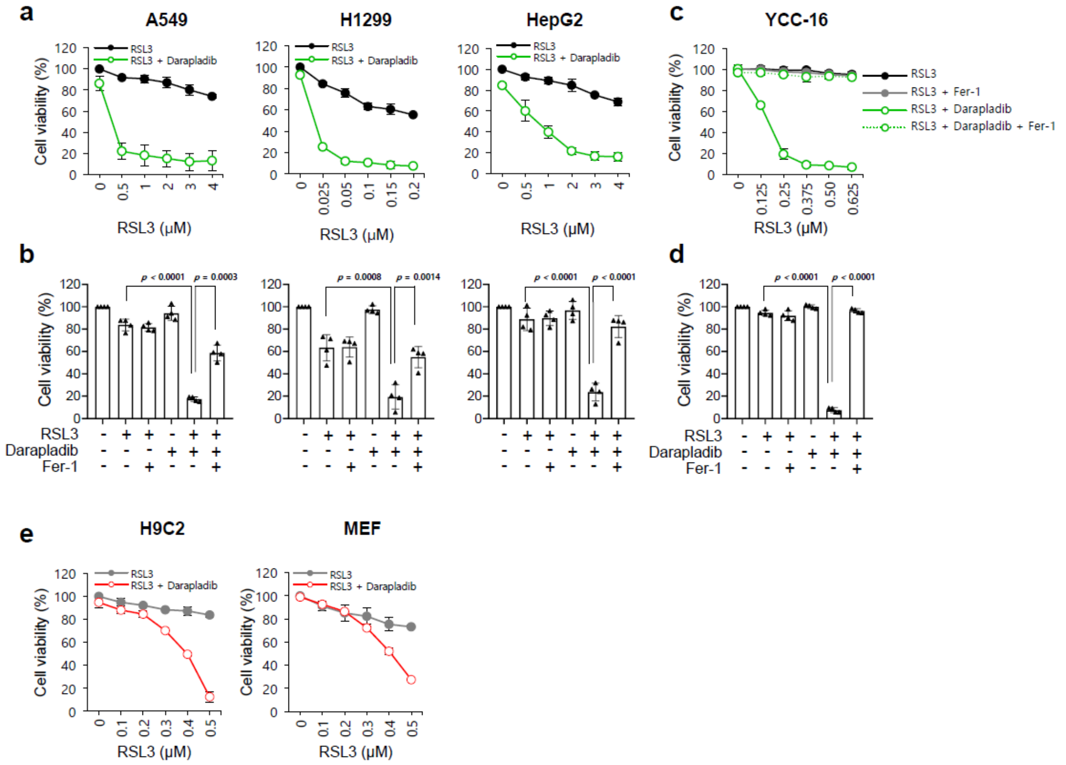

Supplementary Fig. 3. Darapladib sensitises various types of cancer cells to ferroptosis.

(a, b) Relative viability of A549, H1299, and HepG2 cells treated with RSL3 and/or 2 μM darapladib in the presence or absence of Fer-1 for 20 h. The data are presented as the means ± SDs (n = 3 or 4 independent experiments, the significance of the results was assessed using a two-tailed Student' *t* test). (c, d) Relative viability of YCC-16 cells treated with RSL3 and/or 2 μM darapladib in the presence or absence of Fer-1 for 20 h. The data are presented as the means ± SDs (n = 4 independent experiments, the significance of the results was assessed using a two-tailed Student' *t* test). (e) Relative viability of H9c2 and MEF cells treated with RSL3 and/or 2 μM darapladib. The data are presented as the means ± SDs (n = 3 independent experiments). Exact p values provided as source data. Source data are provided as a source data file.

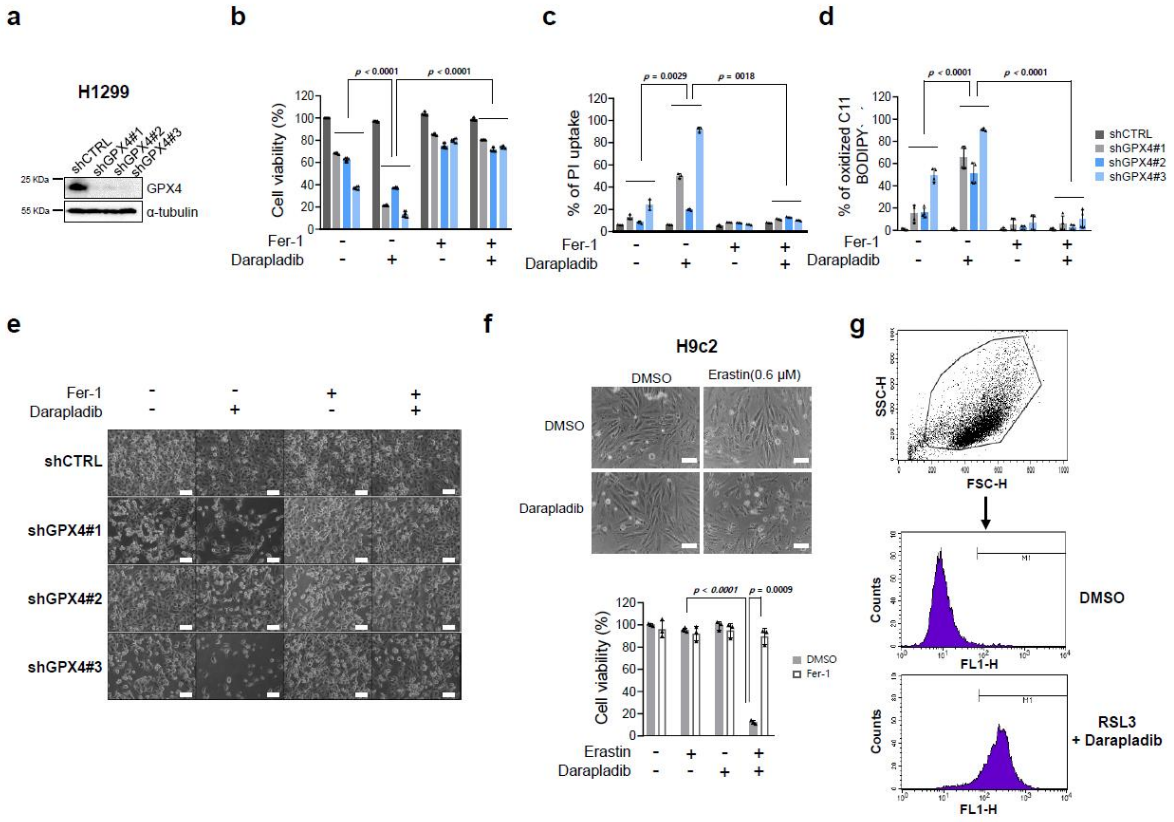

Supplementary Fig. 4. Darapladib augments ferroptosis induced by GPX4 depletion or erastin.

(a) Western blot analysis showing the levels of GPX4 protein in three independent H1299 cells stably transfected with shRNA against GPX4. This experiment was repeated three times. (b-d) Relative cell viability, PI uptake, and lipid peroxidation levels of GPX4-depleted H1299 cells treated with 5 μM darapladib in the presence or absence of 2 μM Fer-1 for 20 h. The data are presented as the means ± SDs (b: n = 8, c: n = 3, d: n = 4 independent experiments, the significance of the results was assessed using a two-tailed Student' *t* test). (e) Images of GPX4-depleted cells treated with 5 μM darapladib and/or 2 μM Fer-1. This experiment was repeated three times. Scale bar, 200 um. (f) Relative viability of H9c2 cells treated with erastin and/or 2 μM darapladib in the presence or absence of Fer-1 for 20 h. The data are presented as the means ± SDs (n = 3 independent experiments, the significance of the results was assessed using a two-tailed Student' *t* test). Scale bar, 200 um. (g) Example gating strategy for flow cytometry analysis of BODIPY-C11 581/591 oxidation in cells. Exact p values provided as source data. Source data are provided as a source data file.

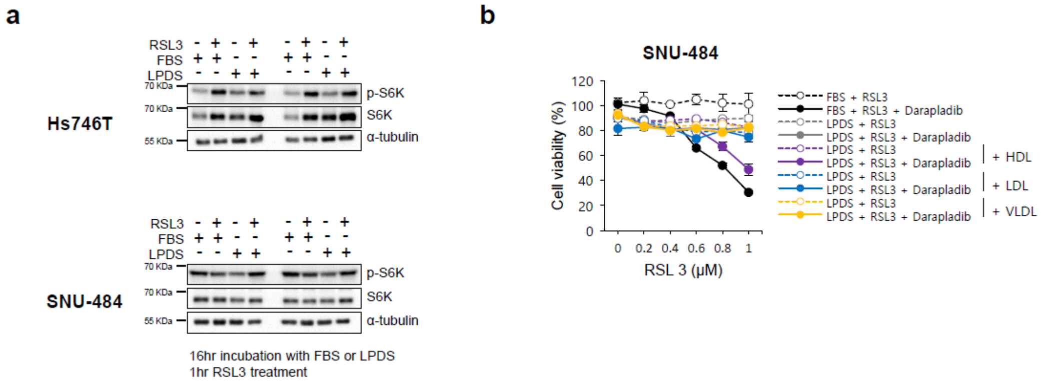

Supplementary Fig. 5. Lipoprotein deficiency delays ferroptosis, possibly due to lack of HDL.

(a) Western blot analysis of p-S6K and S6K to determine whether lipoprotein deficiency affects the mTOR pathway in Hs746T and SNU-484 cells. This experiment was repeated three times. (b) Relative viability of SNU-484 cells cultured in lipoprotein-deficient serum supplemented with HDL, LDL, or VLDL and then treated with RSL3 and 2 μM darapladib for 6 h. The data are presented as the means ± SDs (n = 4 independent experiments). Source data are provided as a source data file.

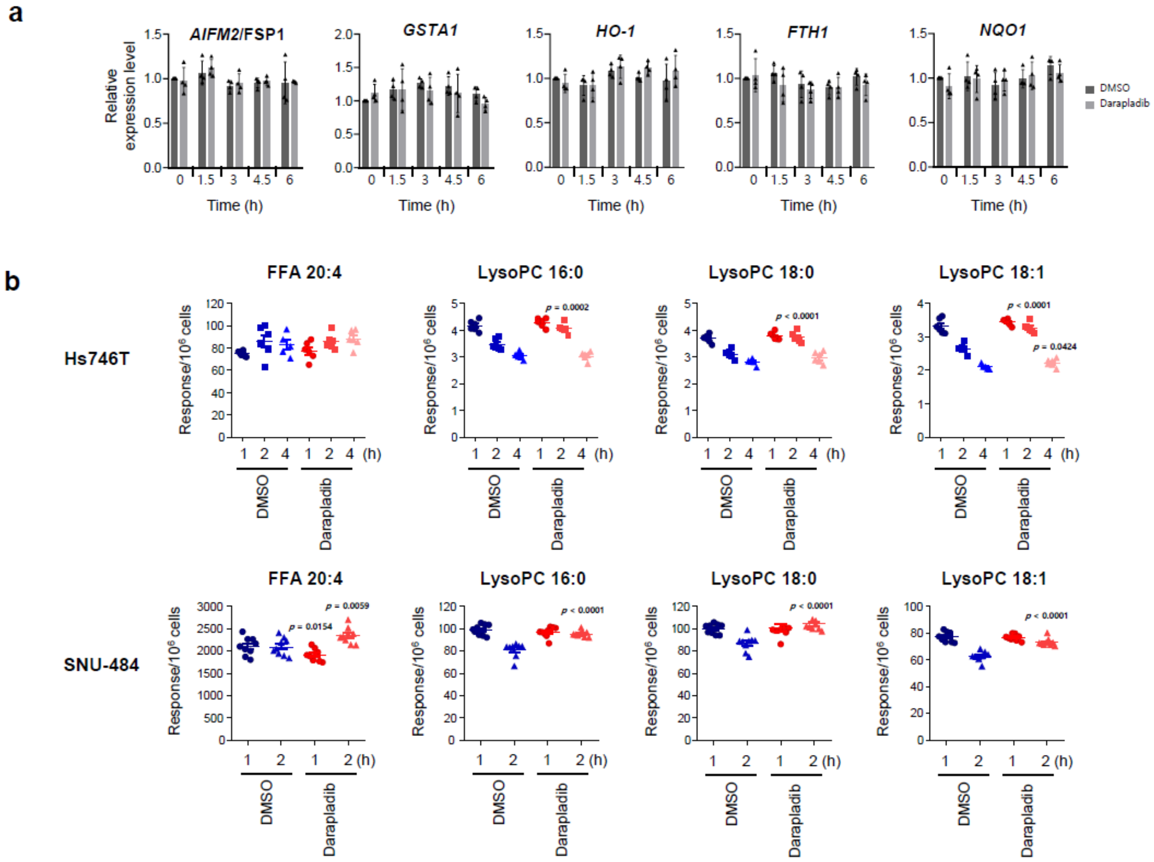

Supplementary Fig. 6. Lp-PLA2 does not contribute to the NRF2 pathway or the extracellular levels of lysoPCs.

(a) Darapladib does not alter NRF2 activity. The mRNA expression levels of NRF2 targets, such as *AIFM2*/FSP1, *GSTA1*, *HO-1*, Fth1 and Nqo1, in Hs746T cells treated with darapladib were analysed. The data are presented as the means ± SDs (n = 4 independent experiments). (b) Levels of AA and lysoPC species in the conditioned medium of Hs746T cells treated with 2 μM darapladib for 0.5, 1, 2, and 4 h determined using LC–MS/MS. The levels of lipids were normalised by the internal standard and cell numbers. The data are presented as the means ± SDs (Hs746T : n = 6, SNU-484 : n = 9 independent experiments, the significance of the results was assessed using two-tailed Student' *t* test). Exact p values provided as source data. Source data are provided as a source data file.

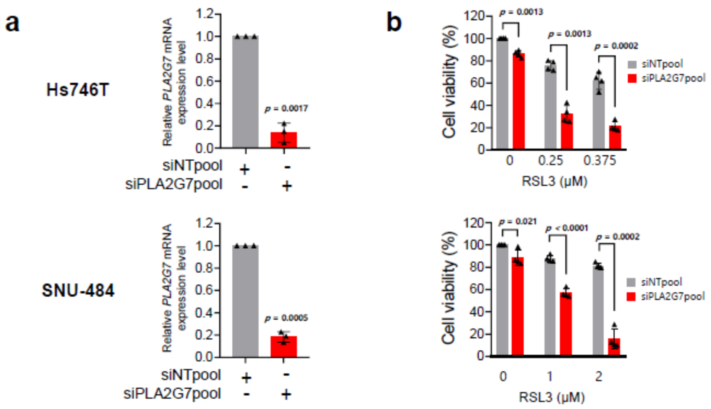

Supplementary Fig. 7. Knockdown of Lp-PLA2 augments RSL3-induced ferroptosis.

(a) Analysis of *PLA2G7* mRNA expression in Hs746T and SNU-484 cells 72 h after siRNA transfection. The data are presented as the means ± SDs (n = 3 independent experiments, the significance of the results was assessed using a two-tailed Student' *t* test). (b) Relative viability of Hs746T and SNU-484 cells treated with RSL3 72 h after siRNA transfection. The relative expression levels were normalised to the β-actin expression levels. The data are presented as the means ± SDs (n = 4 independent experiments, the significance of the results was assessed using a two-tailed Student' *t* test). Exact p values provided as source data. Source data are provided as a source data file.

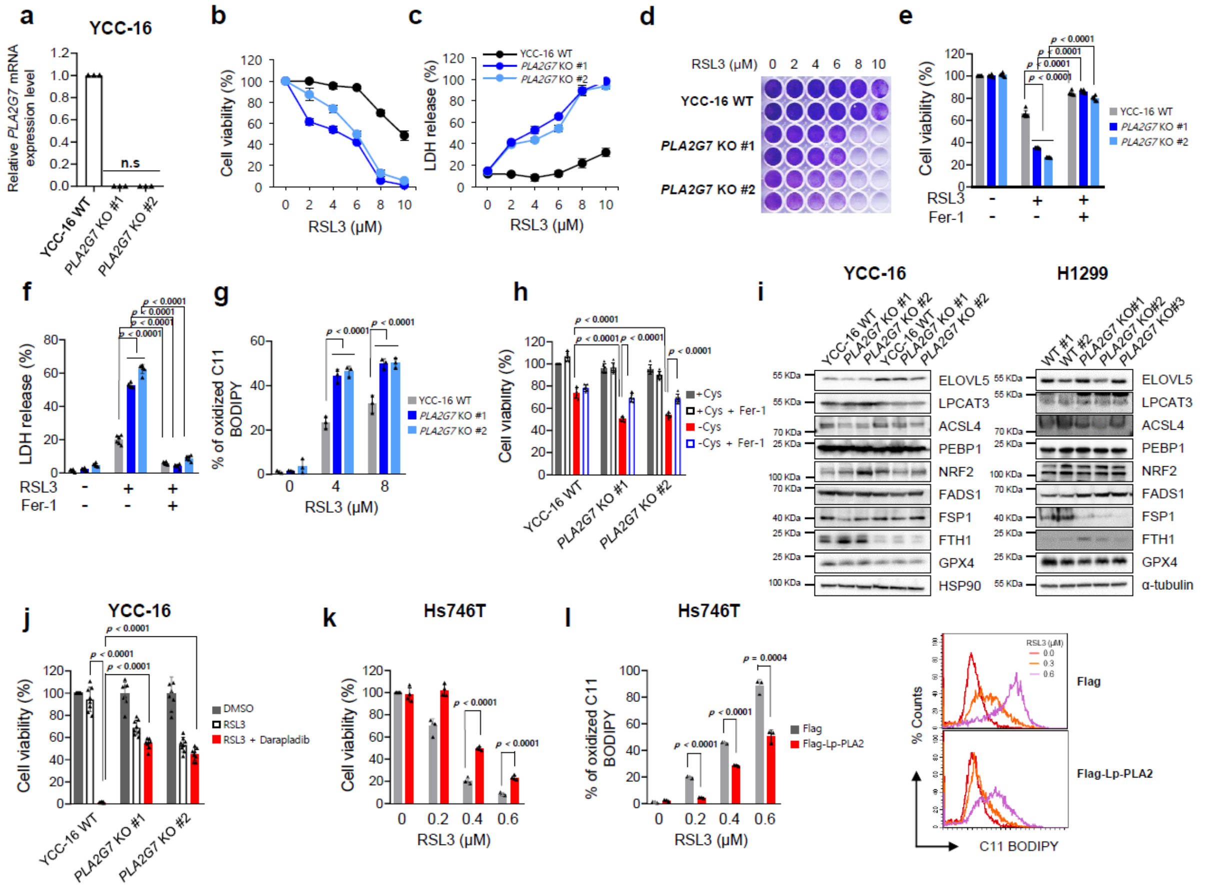

Supplementary Fig. 8. Lp-PLA2 negatively regulates lipid peroxidation and ferroptosis.

(a) Analysis of mRNA expression of *PLA2G7* in *PLA2G7* KO YCC-16 cells normalised to the β-actin expression levels. (n = 3 independent experiments, the significance of the results was assessed using a two-tailed Student' *t* test). (b, c) Relative cell viability and LDH levels of WT and *PLA2G7* KO YCC-16 cells treated with RSL3 for 72 h. The data are presented as the means ± SDs (n = 4 independent experiments). (d) Crystal violet staining of cells treated with increasing concentrations of RSL3. (e, f) Relative cell viability and LDH levels of WT and *PLA2G7* KO YCC-16 cells treated with RSL3 in the presence or absence of Fer-1 for 72 h. The data are presented as the means ± SDs (n = 6 independent experiments, the significance of the results was assessed using a two-tailed Student' *t* test). (g) Lipid peroxidation level of WT and Lp-PLA2 KO YCC-16 cells treated with RSL3 for 2 h. The data are presented as the means ± SDs (n = 3 independent experiments the significance of the results was assessed using a two-tailed Student' *t* test). (h) Viability of WT and Lp-PLA2 KO YCC-16 cells cultured in cysteine-deficient medium for 48 h. The data are presented as the means ± SDs (n = 6 independent experiments, the significance of the results was assessed using a two-tailed Student' *t* test). (i) Western blots

showing the protein expression levels of ferroptosis-related genes in WT and *PLA2G7* KO cells. (j) Relative cell viability of WT and *PLA2G7* KO YCC-16 cells treated with RSL3 and/or darapladib for 48 h. The data are presented as the means ± SDs (n = 8 independent experiments, the significance of the results was assessed using a two-tailed Student' *t* test). (k, l) Relative viability and lipid peroxidation levels of WT and *PLA2G7* KO cells ectopically expressing Lp-PLA2 and treated with RSL3. The data are presented as the means ± SDs (k: n = 4, l: n =3 independent experiments, the significance of the results was assessed using a two-tailed Student' *t* test). Exact p values provided as source data. Source data are provided as a source data file.

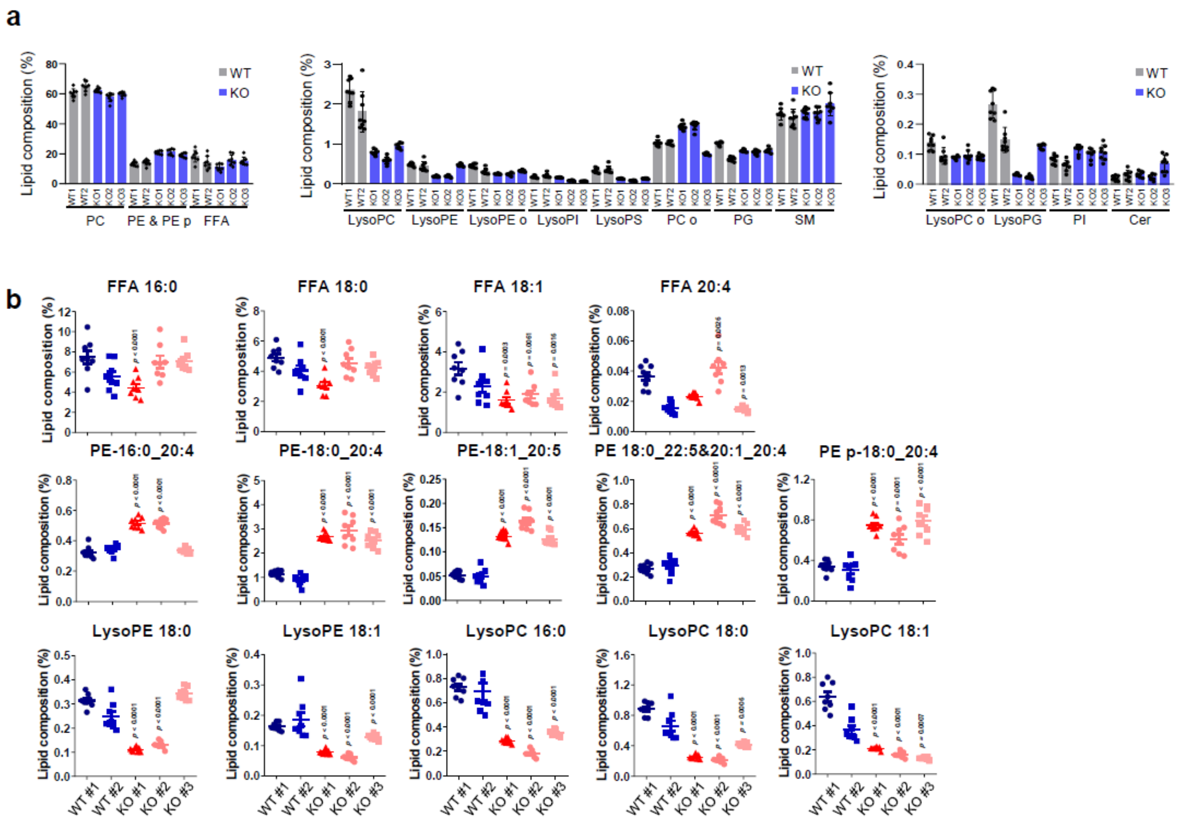

Supplementary Fig. 9. *PLA2G7* deficiency results in the accumulation of PE and PE-p species and the depletion of lysoPE species.

(a) Proportions of various lipid classes normalised by the total lipids detected by LC–MS/MS in WT and *PLA2G7* KO H1299 cells. The y-axis indicates the percentage of the lipid class. The data are presented as the means ± SDs (n = 8 independent experiments). (b) Relative lipid composition of the representative lipid species. The data are presented as the means ± SDs (n =8 independent experiments with, the significance of the results was assessed using one-sided Wilcoxon rank-sum test). Exact p values provided as source data. Source data are provided as a source data file.

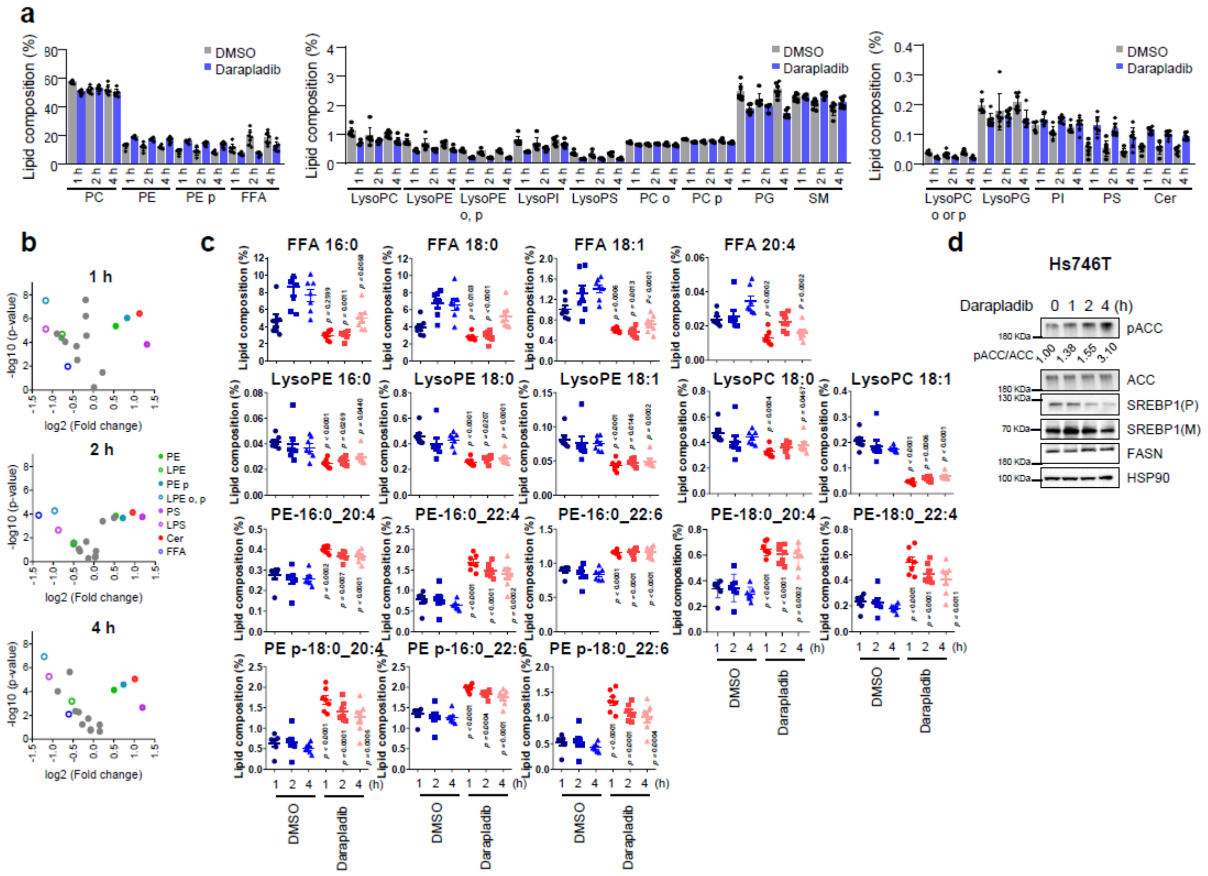

Supplementary Fig. 10. Darapladib induces the accumulation of PE and PE-p species and decreases in lysoPE and MUFA species.

(a) Proportions of various lipid classes normalised by the total lipids detected by LC–MS/MS in Hs746T cells treated with darapladib. The y-axis indicates the percentage of the lipid class. The data are presented as the means ± SDs (n = 7 independent experiments). (b) Volcano plot of lipid classes showing the log2(fold change) and –log10(p) values in the control vs. darapladib-treated Hs746T cells. (c) Levels of representative lipid species in Hs746T cells treated with darapladib for 1, 2, and 4 h were detected by LC–MS/MS. The data are presented as the means ± SDs (n =7 independent experiments with, the significance of the results was assessed using a two-tailed Student' *t* test). (d) Darapladib downregulates the expression of genes involved in de novo fatty acid synthesis. Western blots showing the expression levels of well-known fatty acid metabolism-related genes, such as FASN, ACC, ACC-p, PPAR gamma, and SREBP1, after treatment with darapladib for the indicated times. This experiment was repeated three times. FFA: free fatty acid, Cer: ceramide, SM: sphingomyelin, lysoPC: lysophosphatidylcholine, lysoPE: lysophosphatidylethanolamine, lysoPG: lysophosphatidylglycerol, lysoPI: lysophosphatidylinositol, lysoPS: lysophosphatidylserine, PC: phosphatidylcholine, PE:

phosphatidylethanolamine, PG: phosphatidylglycerol, PI: phosphatidylinositol, PS: phosphatidylserine. Exact p values provided as source data. Source data are provided as a source data file.

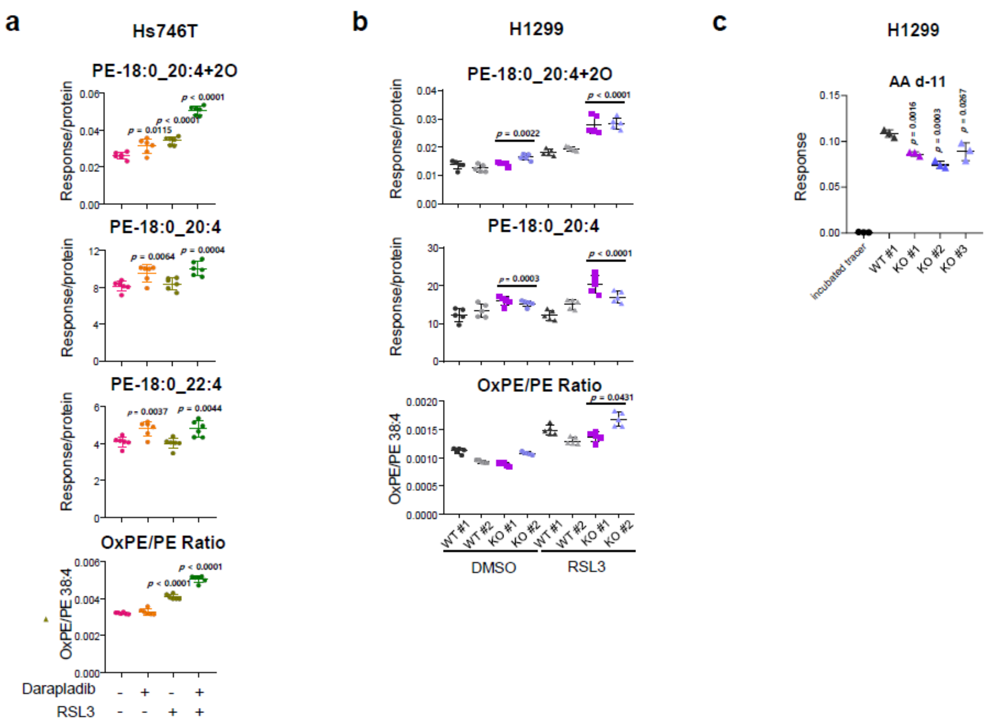

Supplementary Fig. 11. Lp-PLA2 controls both non-oxidised and oxidised PE-18:0/20:4 (SAPE).

(a) Relative levels of oxidised PE-18:0/20:4, PE-18:0_20:4, and PE-18:0_22:4 in Hs746T cells treated with RSL3 and/or 2 μM darapladib. The ratios of oxidised to non-oxidised PE-18:0_20:4 are also shown. The levels of lipids were determined by LC–MS/MS and normalised to the internal standard and cellular protein levels. The data are presented as the means ± SDs (n = 6 independent experiments, the significance of the results was assessed using a two-tailed Student' *t* test). (b) Relative levels of oxidised and non-oxidised PE-18:0_20:4 and their ratio in WT and *PLA2G7* KO H1299 cells treated with RSL3 for 4 h. The data are presented as the means ± SDs (n =5 independent experiments with, the significance of the results was assessed using one-sided Wilcoxon rank-sum test). (c) Analysis of AA-d11 cleavage by PE-18:0_20:4-d11 using lysates from WT and *PLA2G7* KO cells. The data are presented as the means ± SDs (n = 3 independent experiments, the significance of the results was assessed using a two-tailed Student' *t* test). Exact p values provided as source data. Source data are provided as a source data file.

a

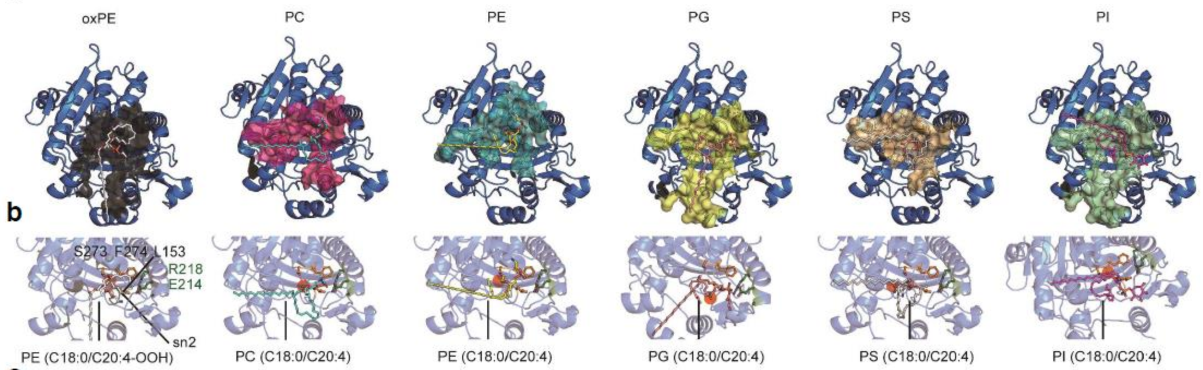

c

Head group	Distance[1] (Å)	Energy (HDOCK)	Rescored energy (kcal/mol)[2]	Calculated Affinity[3]	
oxPE	3.4	-188.92	-5.16	1.63 X 10^{-4} M	
PC	7.0	-160.35	-5.38	1.13 X 10^{-4} M	Zwitterionic at physiological pH
PE	9.4	-178.82	-5.26	1.38 X 10^{-4} M	
PG	8.5	-166.74	-5.34	1.21 X 10^{-4} M	Negatively charged
PS	5.3	-231.29	-5.08	1.87 X 10^{-4} M	
PI	4.6	-193.88	-5.34	1.21 X 10^{-4} M	

[1] Distance between catalytic serine to sn2 of fatty acids
[2] Energies of complex were calculated by PRODIGY
[3] Affinities were calculated based on rescored energy at 298K

Supplementary Fig. 12. Molecular docking analysis of Lp-PLA2 and various phospholipids.

(a) The cartoon representation of the structural docking model of Lp-PLA2 in complex with various phospholipids with different head groups: PC, PE, PS, PI, and PG. The residues involved in interaction in models were represented by surface. The lipid molecules are represented with a ball-and-stick model. The nitrogen and oxygen atoms of lipid molecules are coloured blue and red, respectively. (b) The zoomed view of binding interface between Lp-PLA2 and various phospholipids were represented. The residues involve in known catalysis were coloured red and represented ball and stick model. The residues involved in typical head group interaction were coloured green. (c) Calculated binding affinity between Lp-PLA2 and different phospholipids.

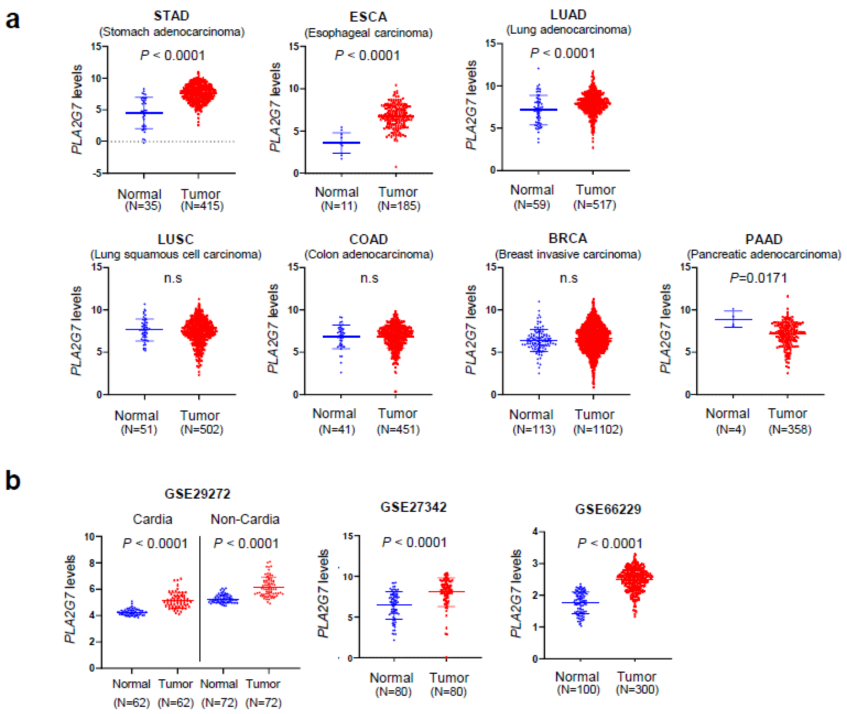

Supplementary Fig. 13. Expression of *PLA2G7* in several types of tumours and normal tissues from transcriptome datasets.

(a) Upregulation of *PLA2G7* in several cancer types, including gastric cancer. Expression of *PLA2G7* in stomach adenocarcinoma (STAD, n_{normal}=35, n_{tumor}=415), oesophageal carcinoma (ESCA, n_{normal}=11, n_{tumor}=185), lung adenocarcinoma (LUAD, n_{normal}=59, n_{tumor}=517), lung squamous cell carcinoma (LUSC, n_{normal}=51, n_{tumor}=502), colon adenocarcinoma (COAD, n_{normal}=41, n_{tumor}=451), breast invasive carcinoma (BRCA, n_{normal}=113, n_{tumor}=1102) and pancreatic adenocarcinoma (PAAD, n_{normal}=4, n_{tumor}=358) tissues and their matched normal tissues. The expression data were extracted from the TCGA repository. (b) Relative expression levels of *PLA2G7* in gastric cancer and normal tissues from independent transcriptome datasets (GSE6622 9: Cardia n_{normal}=62, n_{tumor}=62, Non-cardia n_{normal}=72, n_{tumor}=72, GSE29272 n_{normal}=80, n_{tumor}=80, and GSE27342 n_{normal}=100, n_{tumor}=100). The data are presented as the means ± SDs, the significance of the results was assessed using a two-tailed Student' *t* test). Exact p values provided as source data. Source data are provided as a source data file.

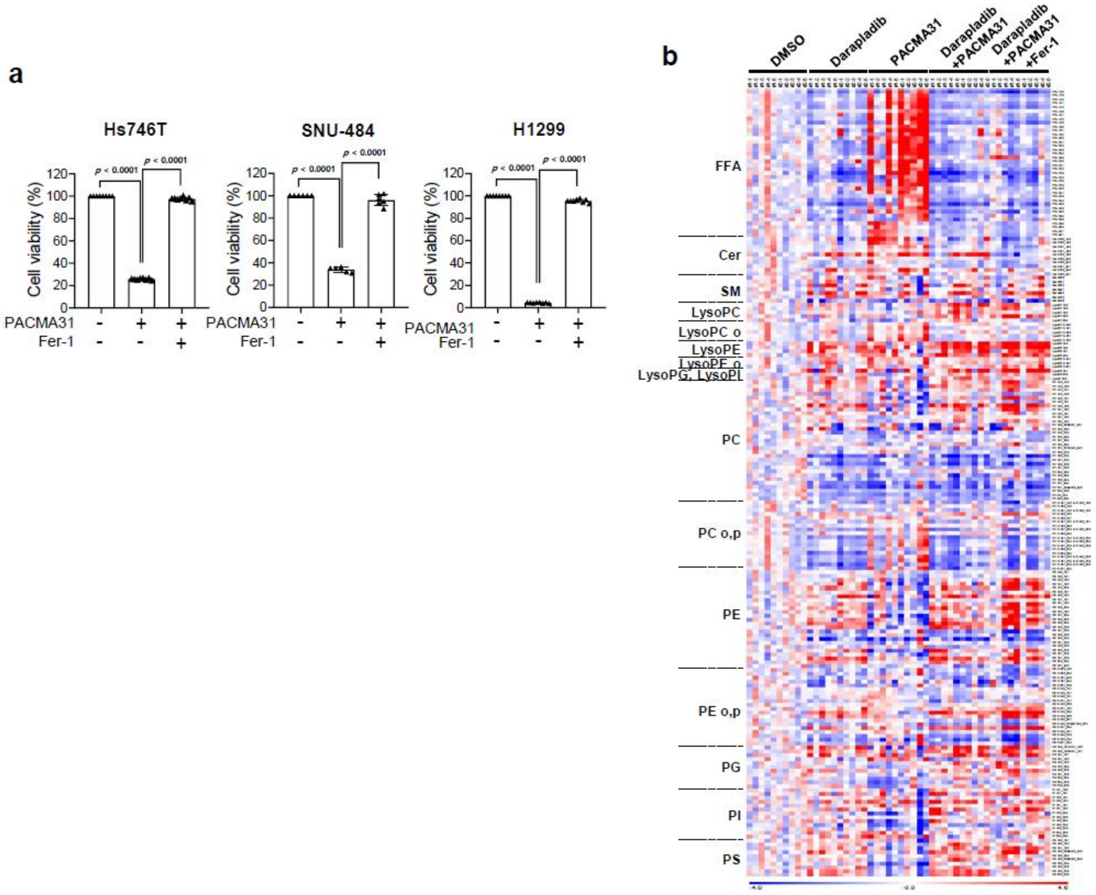

Supplementary Fig. 14. Darapladib induces the accumulation of PE species in xenografted tumour tissues. (a) Relative viability of Hs746T, SNU-484, and H1299 cells treated with PACMA31 in the presence or absence of Fer-1 for 20 h. The data are presented as the means ± SDs (n = 8 or 6 independent experiments, the significance of the results was assessed using a two-tailed Student' *t* test). (b) Relative lipid composition of tumour tissues treated with darapladib, PACMA31, and ferrostatin-1 determined by UPLC/QTOF-MS as described in the legend of Fig. 7g. Exact p values provided as source data. Source data are provided as a source data file.

a

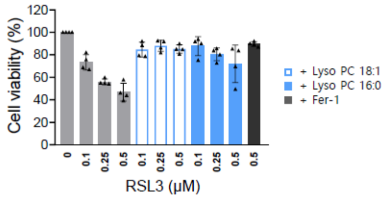

Supplementary Fig. 15. Supplementation with lysoPC18:0 or lysoPC16:0 abrogates RSL3-induced ferroptosis.

(A) Relative viability of Hs746T cells pretreated with LysoPC (16:0 or 18:1) for 1 h and treated with RSL3 and/or 2 μM darapladib for 20 h. The data are presented as the means ± SDs (n = 4 independent experiments). Source data are provided as a source data file.

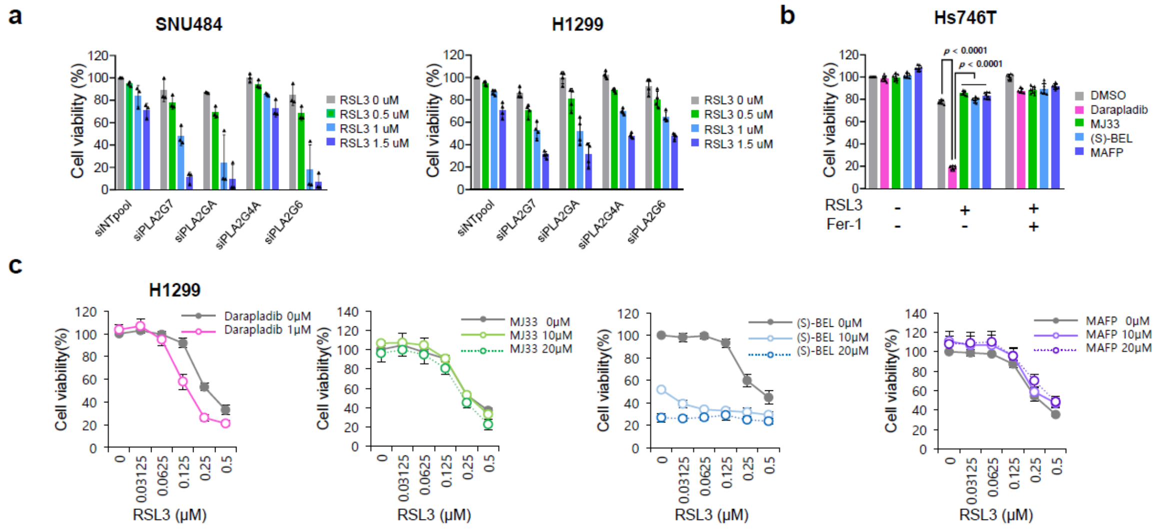

Supplementary Fig. 16. Effect of PLA2 family members on ferroptosis.

(a) Relative viability of SNU-484 and H1299 cells transfected with siRNAs against *PLA2G2A* (sPLA2), *PLA2G4A* (cPLA2), *PLA2G6* (iPLA2), and *PLA2G7* (Lp-PLA2) for 72 and then treated with RSL3. The data are presented as the means ± SDs (SNU-484 : n = 3, H1299: n = 4, independent experiments). (b, c) Relative viability of Hs746T and H1299 cells treated with various PLA2 inhibitor, such as (S)-BEL (an iPLA2 inhibitor), MAFP (a cPLA2/sPLA2 inhibitor), MJ33 (an inhibitor of PLA2 activity of PRDX6) and/or RSL3, in the presence or absence of Fer-1 for 20 h. The data are presented as the means ± SDs (b: n = 8, or c: n = 3 independent experiments, the significance of the results was assessed using a two-tailed Student' *t* test). Exact p values provided as source data. Source data are provided as a source data file.

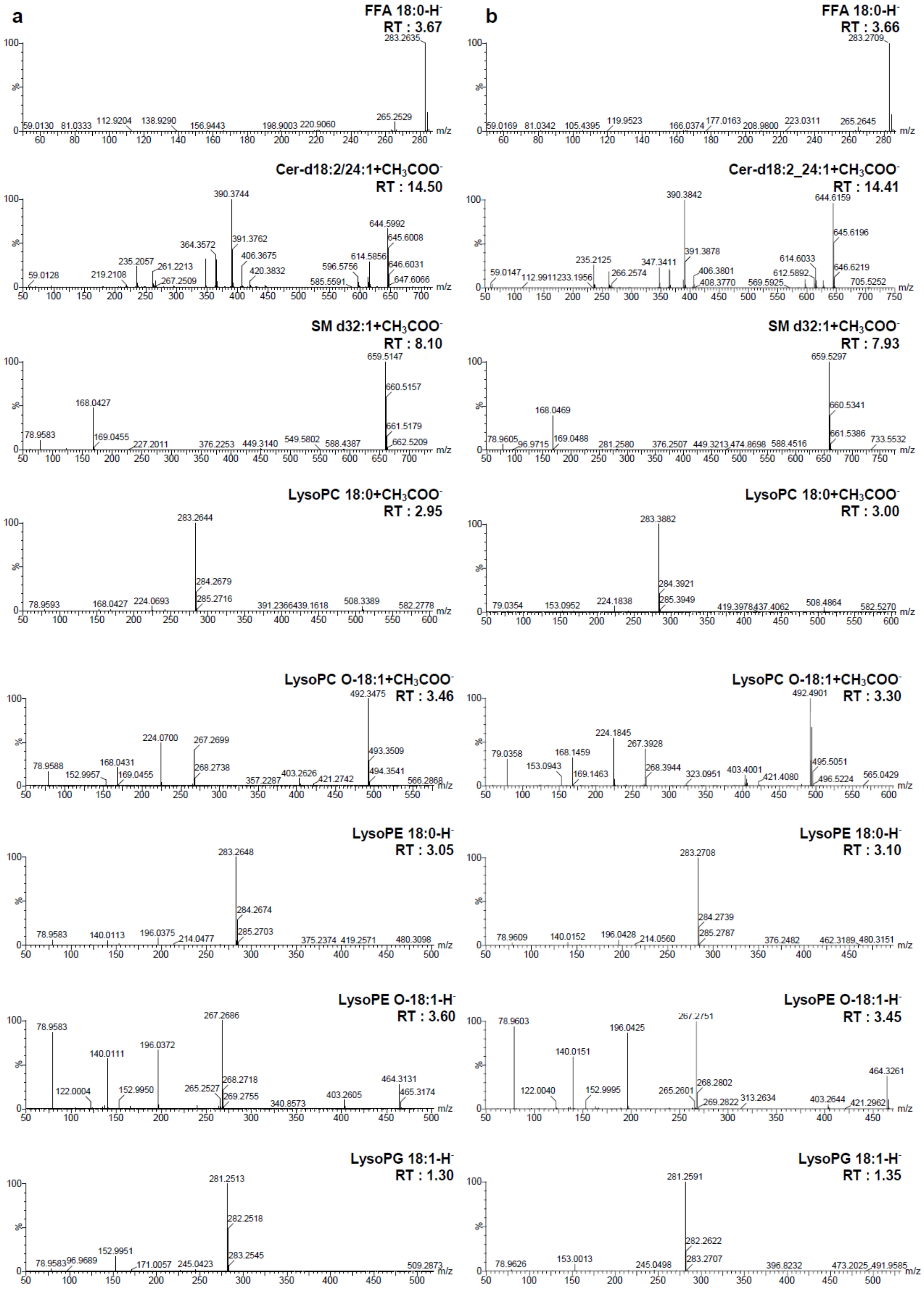

a
FFA 18:0-H⁻
RT : 3.67
283.2635
59.0130 81.0333 112.9204 138.9290 156.9443 198.9003 220.9060 265.2529
Cer-d18:2/24:1+CH3COO⁻
RT : 14.50
390.3744 391.3762 364.3572 406.3675 420.3832 235.2057 261.2213 267.2509 219.2108 59.0128 644.5992 645.6008 614.5856 596.5756 646.6031 585.5591 647.6066
SM d32:1+CH3COO⁻
RT : 8.10
659.5147 660.5157 661.5179 662.5209 168.0427 169.0455 78.9583 227.2011 376.2253 449.3140 549.5802 588.4387
LysoPC 18:0+CH3COO⁻
RT : 2.95
283.2644 284.2679 285.2716 78.9593 168.0427 224.0693 391.2366 439.1618 508.3389 582.2778
LysoPC O-18:1+CH3COO⁻
RT : 3.46
492.3475 493.3509 494.3541 224.0700 267.2699 268.2738 168.0431 169.0455 78.9588 152.9957 357.2287 403.2626 421.2742 566.2868
LysoPE 18:0-H⁻
RT : 3.05
283.2648 284.2674 285.2703 78.9583 140.0113 196.0375 214.0477 375.2374 419.2571 480.3098
LysoPE O-18:1-H⁻
RT : 3.60
267.2686 78.9583 196.0372 140.0111 122.0004 152.9950 265.2527 268.2718 269.2755 340.8573 403.2605 464.3131 465.3174
LysoPG 18:1-H⁻
RT : 1.30
281.2513 282.2518 283.2545 78.9583 96.9689 152.9951 171.0057 245.0423 509.2873
b
FFA 18:0-H⁻
RT : 3.66
283.2709
59.0169 81.0342 105.4395 119.9523 166.0374 177.0163 208.9800 223.0311 265.2645
Cer-d18:2_24:1+CH3COO⁻
RT : 14.41
390.3842 644.6159 645.6196 391.3878 347.3411 235.2125 266.2574 59.0147 112.9911 233.1956 406.3801 408.3770 569.5925 614.6033 612.5892 646.6219 705.5252
SM d32:1+CH3COO⁻
RT : 7.93
659.5297 660.5341 661.5386 733.5532 168.0469 169.0488 78.9605 96.9715 281.2580 376.2507 449.3213 474.8698 588.4516
LysoPC 18:0+CH3COO⁻
RT : 3.00
283.3882 284.3921 285.3949 79.0354 153.0952 224.1838 419.3978 437.4062 508.4864 582.5270
LysoPC O-18:1+CH3COO⁻
RT : 3.30
492.4901 495.5051 496.5224 224.1845 267.3928 268.3944 168.1459 169.1463 79.0358 153.0943 323.0951 403.4001 421.4080 565.0429
LysoPE 18:0-H⁻
RT : 3.10
283.2708 284.2739 285.2787 78.9609 140.0152 196.0428 214.0560 376.2482 462.3189 480.3151
LysoPE O-18:1-H⁻
RT : 3.45
267.2751 78.9603 196.0425 140.0151 122.0040 152.9995 265.2601 268.2802 269.2822 313.2634 403.2644 421.2962 464.3261
LysoPG 18:1-H⁻
RT : 1.35
281.2591 282.2622 283.2707 78.9626 153.0013 245.0498 396.8232 473.2025 491.9585
m/z
%

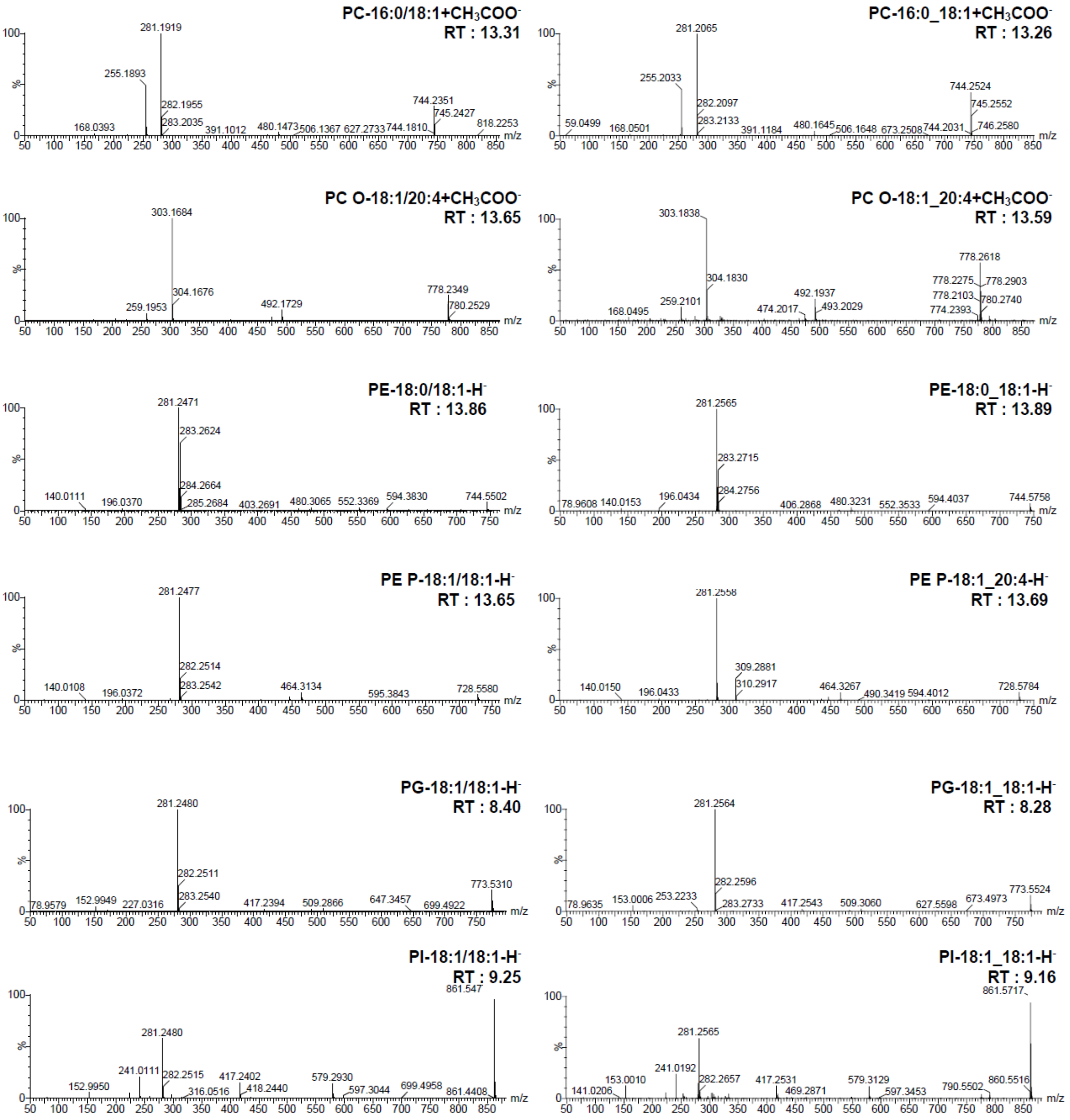

Supplementary Figure 17. Representative MS/MS spectra of (a) lipid standard compounds and (b) tumour tissues acquired from quality control samples.

FFA: free fatty acid, Cer: ceramide, SM: sphingomyelin, lysoPC: lysophosphatidylcholine, lysoPE: lysophosphatidylethanolamine, lysoPG: lysophosphatidylglycerol, PC: phosphatidylcholine, PE: phosphatidylethanolamine, PG: phosphatidylglycerol, PI: phosphatidylinositol.

Supplementary Table 1. MS/MS conditions and retention times of FFA 20:4 and lysoPCs analysed by UPLC-TQ-MS

Name	Polarity	MRM transition (m/z)	Collision energy (eV)	Retention time (min)
FFA 20:4	Negative	303.2 > 303.2	1	4.92
LysoPC 16:0	Negative	554.3 > 255.3	32	5.63
LysoPC 18:0	Negative	582.3 > 283.2	32	7.75
LysoPC 18:1	Negative	580.3 > 281.2	32	5.89
FFA 13:0	Negative	213.2 > 213.2	1	3.58
LysoPC 18:1-d7	Negative	587.4 > 288.2	32	5.89

*** FFA: free fatty acid, lysoPC: lysophosphatidylcholine**

Supplementary Table 2. MRM transition, optimal collision energies, calibration curves, regression coefficients, and dynamic ranges of detection and quantification for the analysis of oxidised PE and PE by UPLC-TQ-MS

name	Polarity	MRM transition (m/z)	Cone voltage (V)	Collision energy (eV)	Retention time (min)	Curve type	Weighting	Calibration curve	r^2	Dynamic range (ppb)
PE-18:0/20:4+2O	Negative	798.5 > 283.2, 798.5 > 317.2	22	36, 24	13.75	Linear	1/x	$y=0.1029*x-0.00399065$	0.999095	0.2-200
PE-18:0/20:4	Negative	766.5 > 283.2, 766.5 > 303.2	30	40, 30	14.72	2nd order	1/x	$y=-6.79887e^{-0.005}*x^2+0.484119*x+5.60103$	0.997819	60-2400
PE-18:0/22:4	Negative	794.5 > 283.2, 794.5 > 331.2	30	40, 30	14.88					
PE-15:0/18:1-d7	Negative	709.5 > 241.2, 709.5 > 288.2	10	40, 30	14.57					

*** PE: phosphatidylethanolamine**

Supplementary Table 3. MS/MS conditions for the detection of FFA 20:4-d11 by UPLC-TQ-MS

name	Polarity	MRM transition (m/z)	Cone voltage (V)	Collision energy (eV)	Retention time (min)
FFA 20:4 m+11	Negative	314.2 > 314.2	20	1	4.53
FFA 13:0	Negative	213.2 > 213.2	20	1	3.30

*** FFA: free fatty acid**

Supplementary Table 4. Materials and antibodies list used in our study.

REAGENT or RESOURCE	SOURCE	IDENTIFIER
Antibodies		
Rabbit polyclonal Anti-ACC	Cell Signaling Technology	3662; RRID: AB_2219400
Rabbit polyclonal Anti-phospho ACC	Cell Signaling Technology	3661; RRID: AB_330377
Rabbit polyclonal Anti- p70 S6 Kinase	Cell Signaling Technology	9202; RRID AB_331676
Rabbit polyclonal Anti-phosphor p70 S6 Kinase	Cell Signaling Technology	9205; RRID AB_330944
Rabbit polyclonal Anti-FASN	Cell Signaling Technology	3180; RRID: AB_2100796
Rabbit polyclonal Anti-FTH1	Cell Signaling Technology	3998; RRID: AB_1903974
Rabbit polyclonal Anti-FADS1	Proteintech	10627-1-AP; RRID: AB_2231403
Rabbit polyclonal Anti-NRF2	Proteintech	16396-1-AP; RRID: AB_ 2782956
Mouse monoclonal Anti-PEBP1	Santa Cruz Biotechnology	sc-376925
Mouse monoclonal Anti-ELOVL5	Santa Cruz Biotechnology	sc-374138; RRID: AB_10920219
Mouse monoclonal Anti-ACSL4	Santa Cruz Biotechnology	Sc-271800; RRID: AB_10715092
Mouse monoclonal Anti-HSP90	Santa Cruz Biotechnology	sc-13119; RRID: AB_ 675659
Mouse monoclonal Anti-FSP1	Santa Cruz Biotechnology	sc-377120; RRID:AB_2893240
Rabbit polyclonal Anti-GPX4	Abcam	Ab125066; RRID: AB_ 10973901
Rabbit polyclonal Anti-Na/K ATPase	Abcam	Ab76020; RRID:AB_1310695
Rabbit polyclonal Anti-LPCAT3	Prosci	16-999
Mouse monoclonal Anti-SREBP1	BD Biosciences	557036; RRID: AB_396559
Mouse monoclonal Anti-alpha-Tubulin	Sigma-Aldrich	T9026; RRID: AB_477593
Mouse monoclonal Anti-FALG	Sigma-Aldrich	F3165; RRID: AB_259529
Mouse monoclonal Anti-beta-Actin	Sigma-Aldrich	A5316; RRID: AB_476743
Goat anti Mouse IgG:HRP	Biorad	103005: RRID:AB_609692
Goat anti Rabbit IgG:HRP	Biorad	403005; RRID:AB_609686
Chemicals, Peptides, and Recombinant Proteins		
RSL3	Selleck Chemicals	S8155; CAS: 1219810-16-8
Ferrostatin-1 (Fer-1)	Selleck Chemicals	S7243; CAS: 347174-05-4
Z-VAD-FMK	Selleck Chemicals	S7023; CAS: 187389-52-2
Darapladib (SB-480848)	Selleck Chemicals	S7520; CAS: 356057-34-6

Erastin	Selleck Chemicals	S7242; CAS: 571203-78-6
ML210	Sigma-Aldrich	SML0521; CAS: 1360705-96-9
Liproxstatin-1	Sigma-Aldrich	SML1414; CAS: 950455-15-9
LDL	Sigma-Aldrich	L7914; CAS: 308068-14-6
HDL	Sigma-Aldrich	L8039; CAS: 308068-14-6
VLDL	Sigma-Aldrich	437647; CAS:
Lipoprotein Deficient Serum from human plasma	Sigma-Aldrich	S5519; CAS
PACMA31	Tocris	5116; CAS: 1401089-31-3
Necrostatin-1	Enzo Life Sciences	BML-AP309; CAS: 4311-88-0
MAFP	Cayman Chemical	70660 CAS: 188404-10-6
(S)-BEL (Bromoenol lactone)	Cayman Chemical	70700 CAS: 88070-98-8
MJ33 (lithium salt)	Cayman Chemical	90001844 CAS: 1007476-63-2
JKE1674	Cayman Chemical	30784 CAS: 2421119-60-8
Oleic acid	Cayman Chemical	90260; CAS: 112-80-1
Arachidonic Acid	Cayman Chemical	90010; CAS: 506-32-1
1-Stearoyl-2- Arachidonyl-d11-sn-glycero-3-PE (SAPE-d11)	Cayman Chemical	27929; CAS: 2750554-96-0
SPLASH Lipidomix	Avanti Polar Lipids	330707; CAS: 67-56-1
Tridecanoic acid	Sigma-Aldrich	91988; CAS: 638-53-9
1-stearoyl-2-arachidonoyl-sn-glycero-3-PE (PE 18:0_20:4)	Sigma-Aldrich	850804C;CAS: 61216-62-4
Critical Commercial Assays		
BODIPY 581/591 C11 (Lipid Peroxidation Sensor)	Invitrogen	D3861
CellTiter-Glo 2.0 Cell Viability Assay	Promega	G9243
Cytotoxicity Detection Kit (LDH)	Roche	11644793001
Iron assay kit	Sigma-Aldrich	MAK025
Deposited Data		
TCGA Pan-Cancer (PANCAN) dataset		http://xena.ucsc.edu
Experimental Models: Cell Lines		
Human: Hs 746T	KCLB	Cat# 30135; RRID: CVCL_0333
Human: SNU-484	KCLB	Cat# 00484; RRID: CVCL_0100
Human:YCC-16	KCLB	RRID: CVCL_9649
Human:H1299	ATCC	Cat# CRL-5803; RRID: CVCL_0060
Human:A549	ATCC	Cat# CCL-185; RRID: CVCL_0023
Human:HepG2	ATCC	Cat# HB-8065; RRID: CVCL_0027

Human:H9c2	ATCC	Cat# CRL-1446; RRID: CVCL_0286
Human:HEK293T	ATCC	Cat# CRL-3216; RRID: CVCL_0063
Oligonucleotides		
Primers for qPCR, see method section	This paper	N/A
Software and Algorithms		
Prism 8	GraphPad Software	https://www.graphpad.com/scientific-software/prism/; RRID: SCR_002798
Excel		
PyMOL software (version 1.8)		http://www.pymol.org; RRID:SCR_000305
MarvinView software (version 23.5.0)		https://docs.chemaxon.com/display/lts-mercury/compound-registration-history-of-changes.md
Modeller software (version 10.4)		http://salilab.org/modeller/modeller.html; RRID:SCR_008395
Chemaxon (version 20.8.5)		https://www.chemaxon.com RRID:SCR_004111
AutoDock Vina (version 1.2.0)		http://vina.scripps.ed RRID:SCR_011958
Progenesis QI software (version 2.0)		http://www.nonlinear.com/progenesis/qi-for-proteomics/ RRID:SCR_018923
MarkerViewTM software (version 1.3.1)		https://sciex.com/products/software/markerview-software
Mass Hunter Workstation (version B.06.00)		https://www.agilent.com/en/support/software-informatics/qual-b-0600-sp1
BD CellQuest Pro (version 5. 1)		https://www.bdbiosciences.com/documents/15_cellquest_prosoft_analysis.pdf RRID:SCR_014489
MassLynx (version 4.2)		http://www.waters.com/waters/en_US/MassLynx-MS-Software/nav.htm?cid=513662&locale=en_US RRID:SCR_014271
Other		
DMEM	Hyclone	Cat# SH30243.01
MEM	Hyclone	Cat# SH30024.01
RPMI1640	Corning	Cat# 10-040-CVRC

DMEM	Gibco	Cat# 21013024
L-Glutamine	Gibco	Cat# 25030-081
Fetal bovine serum	Gibco	Cat# 10099-141
Fetal bovine serum, dialyzed	Gibco	Cat# 26400-044
Antibiotic-Antimyocotic, 100X	Gibco	Cat# 15240-062
Puromycin Dihydrochloride	Gibco	Cat# A1113803
Propidium Iodide	Inivtrogen	Cat# P1304MP
Lipofectamine™ RNAiMAX transfection reagent	Inivtrogen	Cat# 13778075
Lipofectamine™ 3000 Transfection Reagent	Inivtrogen	Cat# L3000075

Supplementary Figure. 4 a (uncropped scans)

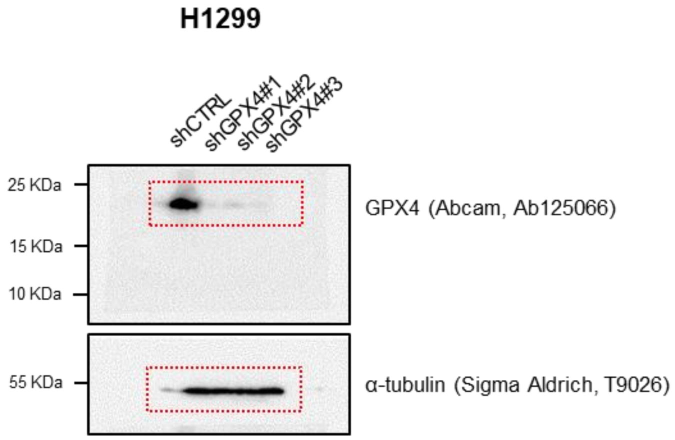

Supplementary Figure. 5 a (uncropped scans)

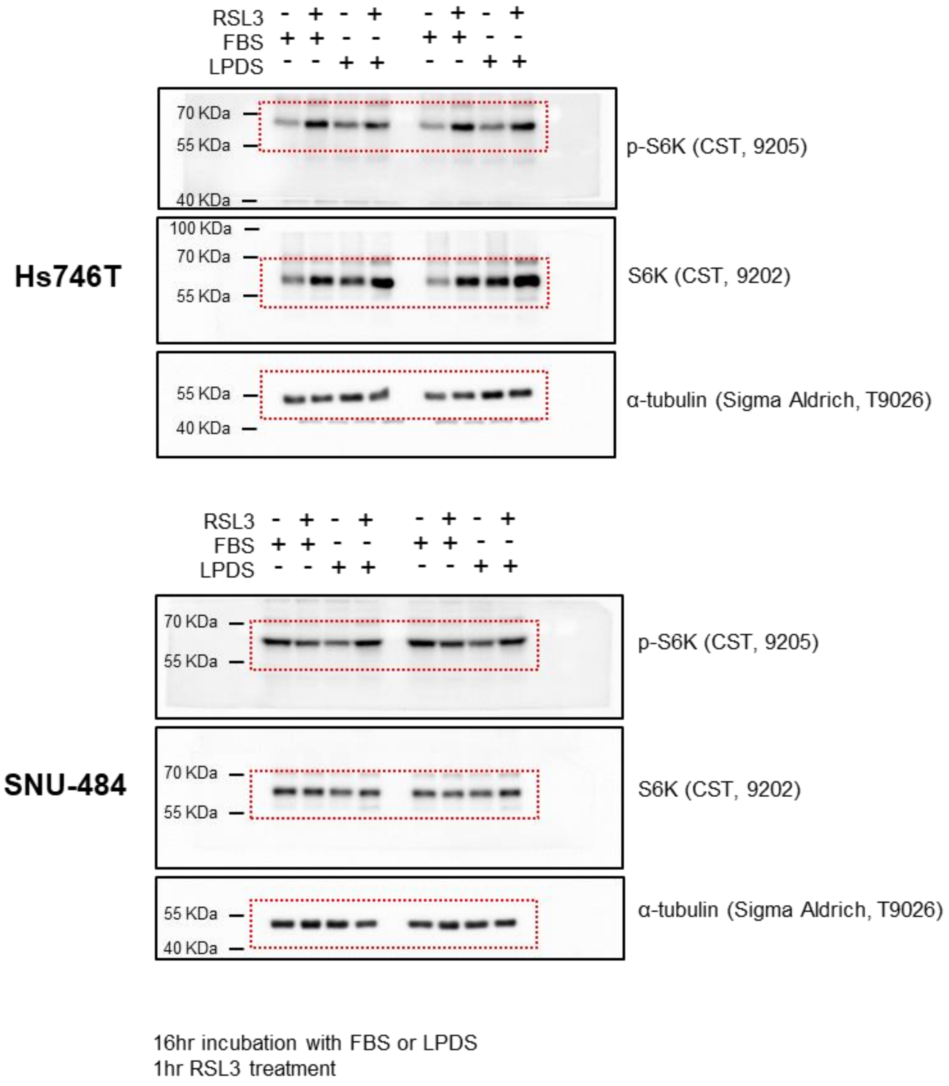

Supplementary Figure. 8 l (uncropped scans)

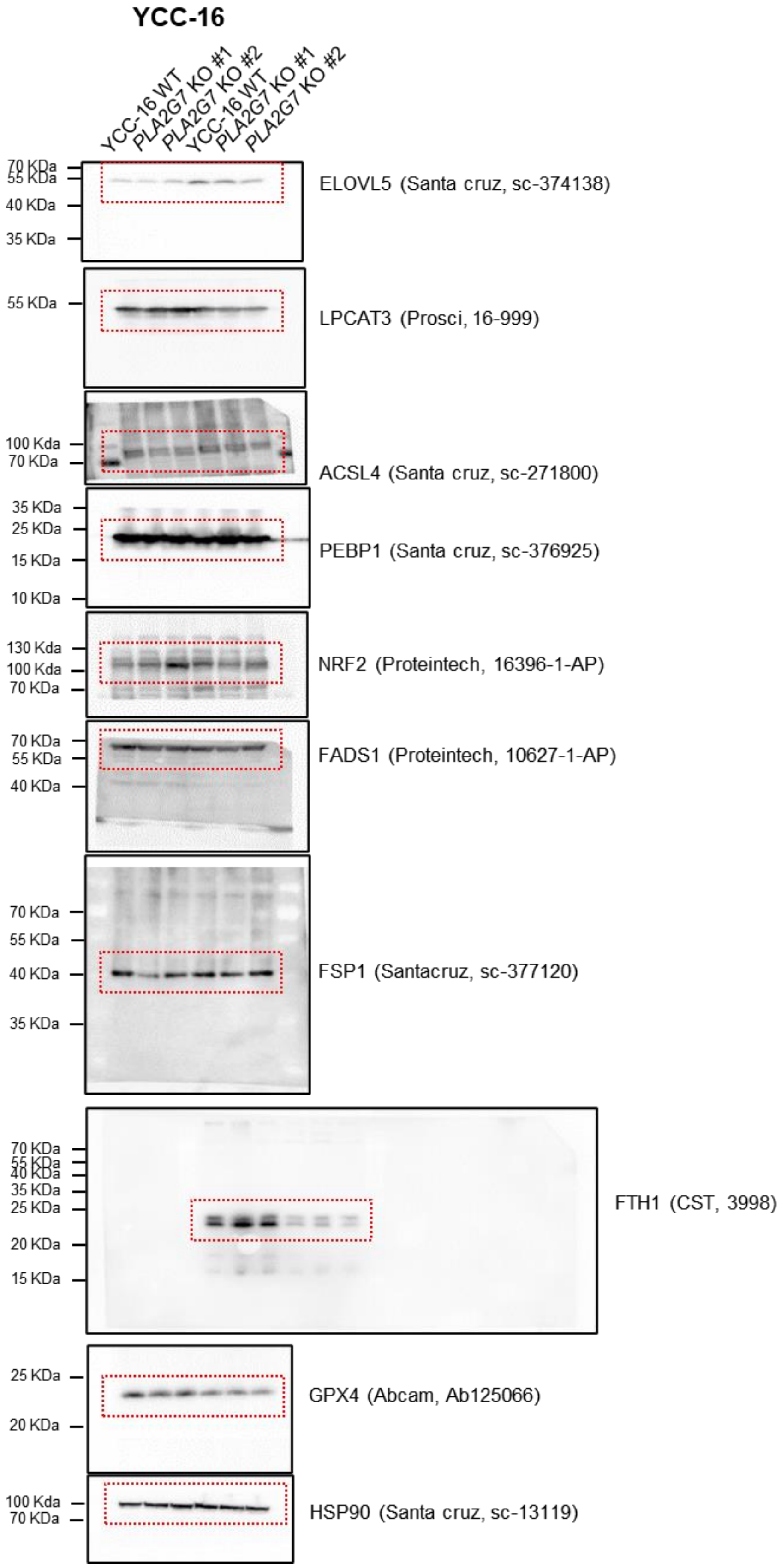

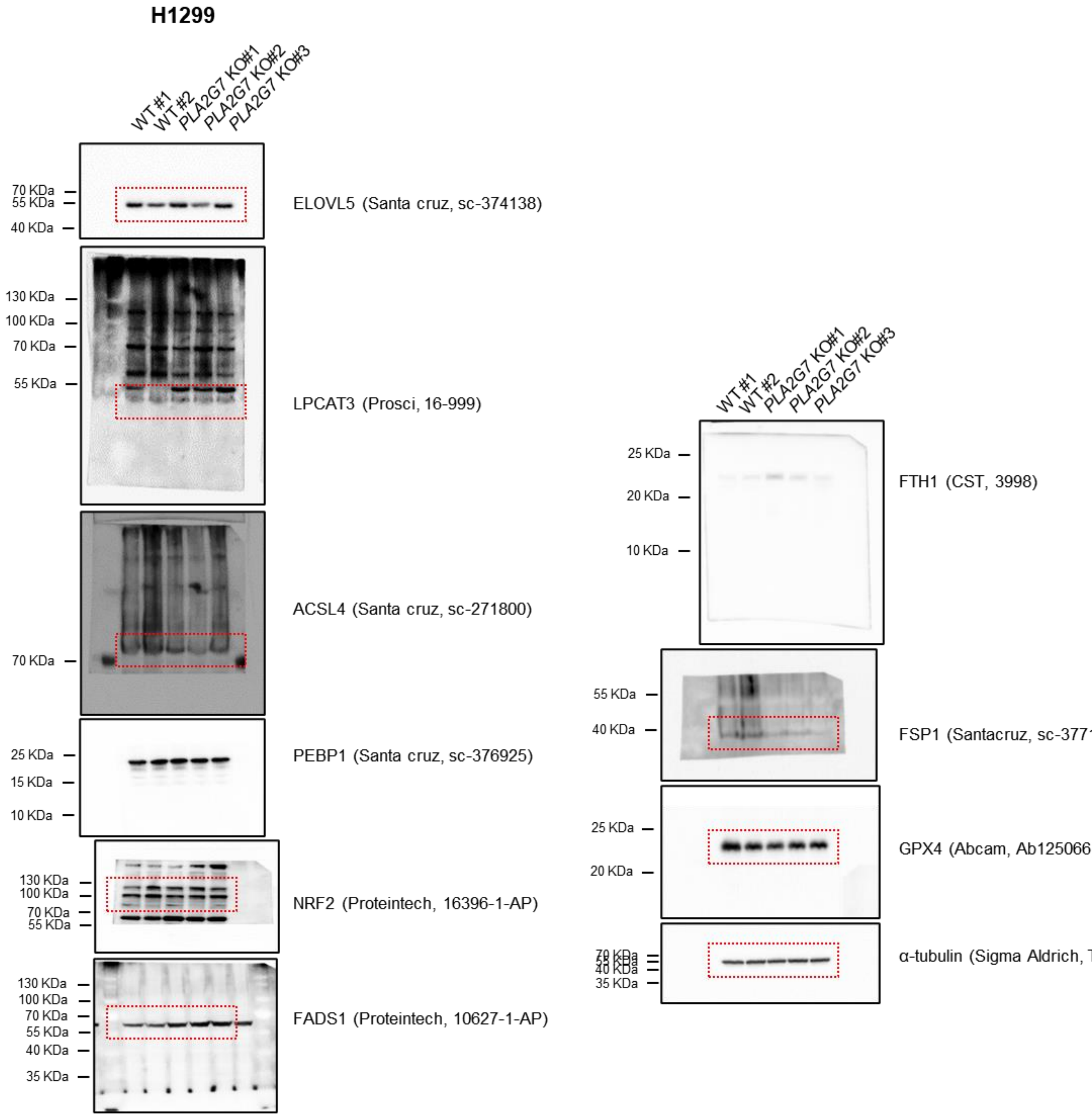
H1299
WT #1
WT #2
PLA2G7 KO#1
PLA2G7 KO#2
PLA2G7 KO#3
70 KDa
55 KDa
40 KDa
ELOVL5 (Santa cruz, sc-374138)
130 KDa
100 KDa
70 KDa
55 KDa
LPCAT3 (Prosci, 16-999)
ACSL4 (Santa cruz, sc-271800)
70 KDa
25 KDa
15 KDa
10 KDa
PEBP1 (Santa cruz, sc-376925)
130 KDa
100 KDa
70 KDa
55 KDa
NRF2 (Proteintech, 16396-1-AP)
130 KDa
100 KDa
70 KDa
55 KDa
40 KDa
35 KDa
FADS1 (Proteintech, 10627-1-AP)
WT #1
WT #2
PLA2G7 KO#1
PLA2G7 KO#2
PLA2G7 KO#3
25 KDa
20 KDa
10 KDa
FTH1 (CST, 3998)
55 KDa
40 KDa
FSP1 (Santacruz, sc-377
25 KDa
20 KDa
GPX4 (Abcam, Ab125066
35 KDa
α-tubulin (Sigma Aldrich,

Supplementary Figure. 10 d (uncropped scans)

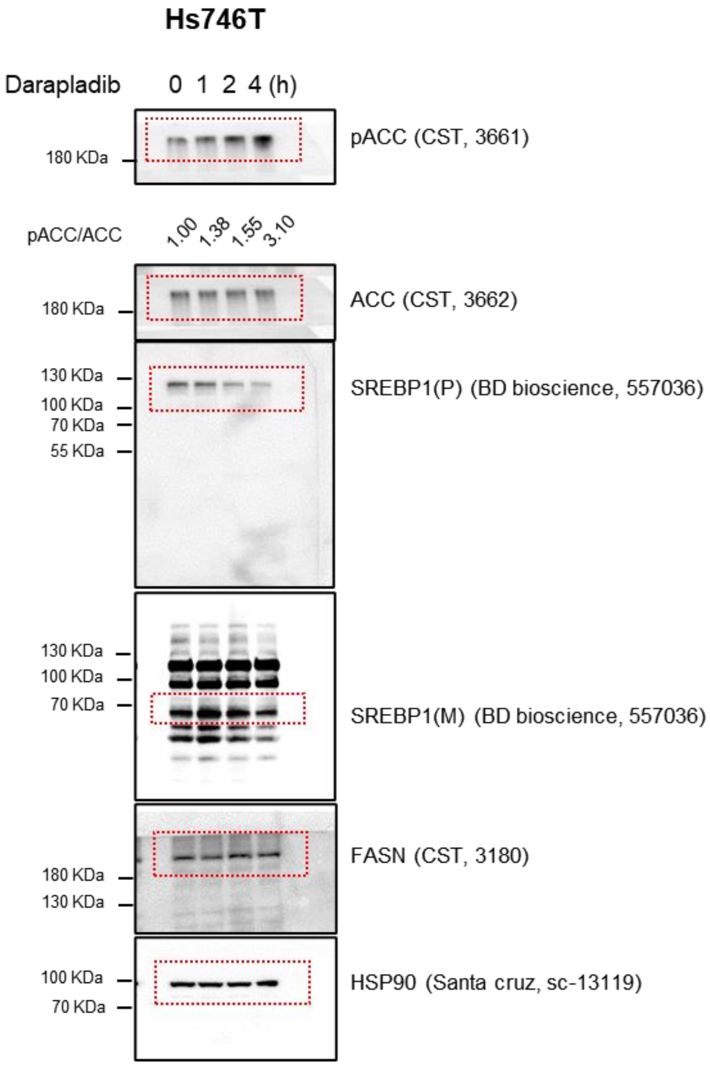

Supplement: Supplementary file 11 — Supplementary Information [file 41467_2023_41462_MOESM11_ESM.pdf]
