# Supplementary material for: The lipoprotein-associated phospholipase A2 inhibitor Darapladib sensitises cancer cells to ferroptosis by remodelling lipid metabolism
Source: Nat Commun. 2023 Sep 15;14:5728. doi: 10.1038/s41467-023-41462-9 (PMC10504358; doi:10.1038/s41467-023-41462-9)
Supplement: Supplementary file 12 — Description of Additional Supplementary files [file 41467_2023_41462_MOESM12_ESM.pdf]

Description of additional supplementary files

Supplementary data 1.
Lipidomic analysis acquired from the UPLC/QTOF MS spectra of WT and PLA2G7 KO H1299 cells.

Supplementary data 2.
Lipidomic analysis based on the UPLC/QTOF MS spectra of Hs746T cells treated with 2 μM darapladib for 1, 2, and 4 h.

Supplementary data 3.
Concentrations of oxidised PE-18:0_20:4, PE-18:0_20:4, and PE-18:0_22:4 in Hs746T cells treatment with RSL3 and/or 2 μM darapladib for 4 h and in SNU-484 cells after treatment with RSL3 and/or 2 μM darapladib for 3 h. The ratios of oxidised to non-oxidised PE-18:0_20:4 are also shown. The concentrations were determined by LC–MS/MS and were normalised to the cellular protein level.

Supplementary data 4.
PE-18:0_20:4-d11 cleavage analysis of H1299 cell lysates incubated with the indicated concentrations of darapladib and (S)-BEL for 3 h and subjected to detection of AA-d11 by LC–MS/MS.

Supplementary data 5.
Levels of AA and lysoPC species in the conditioned medium of Hs746T cells treated with 2 μM darapladib for 0.5, 1, 2, and 4 h determined using LC–MS/MS.

Supplementary data 6.
Relative levels of oxidised PE-18:0/20:4, PE-18:0_20:4, and PE-18:0_22:4 in Hs746T cells treated with RSL3 and/or 2 μM darapladib. The ratios of oxidised to non-oxidised PE-18:0_20:4 are also shown. The levels of lipids were determined by LC–MS/MS and normalised to the internal standard and cellular protein levels.

Supplementary data 7.
Relative levels of oxidised and non-oxidised PE-18:0_20:4 and their ratio in WT and PLA2G7 KO H1299 cells treated with RSL3 for 4 h.

Supplementary data 8.
Analysis of AA-d11 cleavage by PE-18:0_20:4-d11 using lysates from WT and PLA2G7 KO H1299 cells.

Supplementary data 9.
Relative lipid composition of tumour tissues treated with darapladib, PACMA31, and ferrostatin-1 determined by UPLC/QTOF-MS
